# Docking of Secretory Vesicles Is Syntaxin Dependent

**DOI:** 10.1371/journal.pone.0000126

**Published:** 2006-12-27

**Authors:** Heidi de Wit, L. Niels Cornelisse, Ruud F.G. Toonen, Matthijs Verhage

**Affiliations:** Department of Functional Genomics, Center for Neurogenomics and Cognitive Research, Vrije Universiteit Amsterdam and VU Medical Center, Amsterdam, The Netherlands; University of Geveva, Switzerland

## Abstract

Secretory vesicles dock at the plasma membrane before they undergo fusion. Molecular docking mechanisms are poorly defined but believed to be independent of SNARE proteins. Here, we challenged this hypothesis by acute deletion of the target SNARE, syntaxin, in vertebrate neurons and neuroendocrine cells. Deletion resulted in fusion arrest in both systems. No docking defects were observed in synapses, in line with previous observations. However, a drastic reduction in morphologically docked secretory vesicles was observed in chromaffin cells. Syntaxin-deficient chromaffin cells showed a small reduction in total and plasma membrane staining for the docking factor Munc18-1, which appears insufficient to explain the drastic reduction in docking. The sub-membrane cortical actin network was unaffected by syntaxin deletion. These observations expose a docking role for syntaxin in the neuroendocrine system. Additional layers of regulation may have evolved to make syntaxin redundant for docking in highly specialized systems like synaptic active zones.

## Introduction

Intracellular transport vesicles dock at the target membrane prior to fusion. Docking is morphologically well defined, but the molecular mechanisms are poorly understood. Based on experiments in mammalian synaptosomes and by analogy to vesicle trafficking in yeast, SNARE (soluble NSF-attachment protein receptor) proteins were proposed to achieve docking by assembly of a complex consisting of the v-SNARE synaptobrevin and the plasma membrane target SNAREs (t-SNAREs), syntaxin and SNAP-25 (25 kDa synaptosomal-associated protein) [1], [2]. However, due to a large number of interference studies in squid synapses, *Drosophila* neuromuscular junctions, and mouse synapses and chromaffin cells, it has been concluded that SNARE complex assembly occurs downstream of docking [3]–[9]. On the other hand, several observations *in vitro* continue to make a docking role plausible, at least for t-SNAREs [10], [11] and vesicles appear to dock preferentially in t-SNARE-rich membrane patches [12]. Moreover, despite a wide variety of interference studies on presynaptic proteins, only a few subtle alterations in docking have been observed in synapses to suggest alternatives for SNARE dependent docking [13]–[16]. Hence, unlike for other steps in the synaptic vesicle cycle, the docking step remains elusive with no consistent working model and plausible candidate genes.

Munc18-1, a hydrophilic protein with no inherent affinity for membranes, interacts with the t-SNARE syntaxin [17], [18] (for review see [19]) and (*m)unc18-1* null alleles produced a drastic docking defect in mouse chromaffin cells [20] and a mild docking defect in nematode neuromuscular junctions [16], but not in mouse embryonic central nervous system (CNS) synapses [21]. The docking role of Munc18-1 may depend on its syntaxin interaction. Expression of syntaxin binding mutants of Munc18-1 reduced its plasma membrane association [22] and overexpression of yeast syntaxins Sso1p and Sso2p suppressed the secretion defect in yeast mutants deficient for the Munc18-1 ortholog, Sec1p [23]. For these reasons, we proposed that the Munc18-1/syntaxin dimer functions as a docking platform, at least in neurosecretory cells [20]. In agreement with this, we observed increased vesicle docking after overexpression of Munc18-1 in both neurosecretory cells [24] and CNS synapses (RFGT et al., in preparation). These considerations strengthen the suggestion that the t-SNARE syntaxin may be important in secretory vesicle docking, but direct evidence is lacking.

The aim of this study was to test the role of syntaxin in vesicle docking in both neurosecretory cells and synapses by deleting syntaxins 1, 2 and 3 via acute viral expression of Botulinium neurotoxin serotype C (BoNT/C) light chain [25], [26] and morphometric analysis of docking at the ultra structural level. Syntaxin deletion resulted in secretion defects and a robust reduction of docking in neurosecretory cells, but not in CNS synapses. We argue that syntaxin is a bona vide docking factor that may have become redundant in highly specialized systems like CNS active zones.

## Results

### Impaired secretory vesicle docking after syntaxin deletion

**Figure 1:**
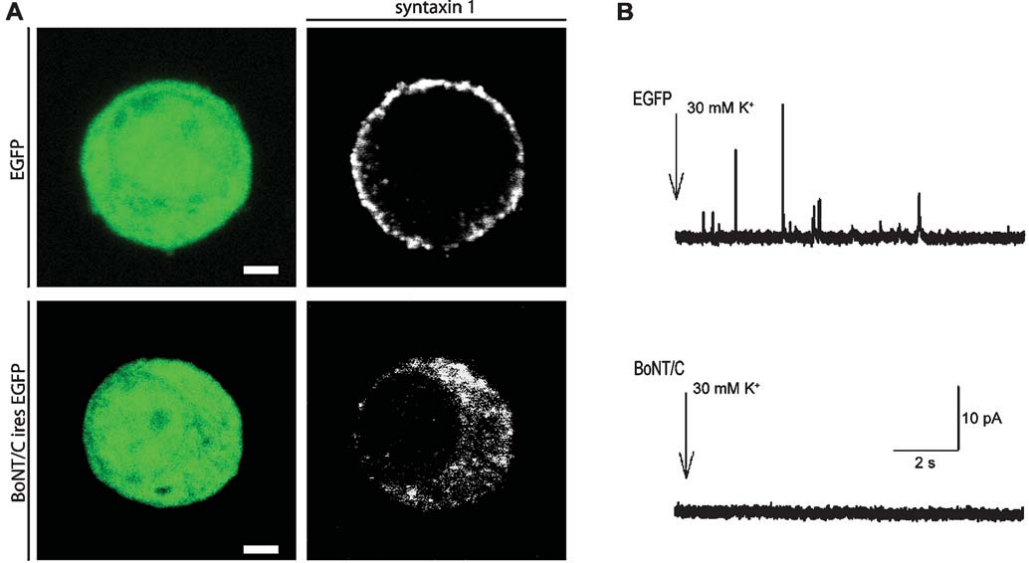
Evoked catecholeamine release is absent in syntaxin deleted chromaffin cells. (A) Fluorescent image of cultured chromaffin cells incubated with SFV-*egfp* or SFV *BoNT/C*-ires-*egfp*, and immunostained for syntaxin, showing a reduced syntaxin staining at the plasma membrane after BoNT/C expression. The syntaxin staining after BoNT/C was slightly overexposed to emphasize the persistence of cytosolic staining (as opposed to plasma membrane staining). Scale bars represent 2 µm. (B) Examples of amperometric recordings in control and BoNT/C infected chromaffin cells during stimulation with a 30 mM K^+^ solution.

To delete syntaxin in (E18) chromaffin cells we expressed BoNT/C light chain from a bicistronic message containing enhanced green fluorescent protein (*egfp*) using the Semliki forest virus (SFV) expression system [24]. In control chromaffin cells, endogenous syntaxin 1 localized in defined clusters at the plasma membrane, in agreement with previous studies [12], [27], and many amperometric spikes were induced by high potassium stimulation (Figure 1B). Syntaxin1 staining revealed a major reduction at the plasma membrane after 6 hours of SFV BoNT/C infection (Figure 1A), and no secretion events were induced by stimulation (Figure 1B), as shown before [28]. A low cytoplasmic syntaxin staining remained after BoNT/C proteolysis (Figure 1A, see also Figure 5A) probably because the HPC1 antibody still recognizes the proteolysed protein. The cytoplasmic levels of this (soluble) protein may be low due to non-specific degradation and/or may be lost during permeabilization in preparation for staining.

**Figure 2:**
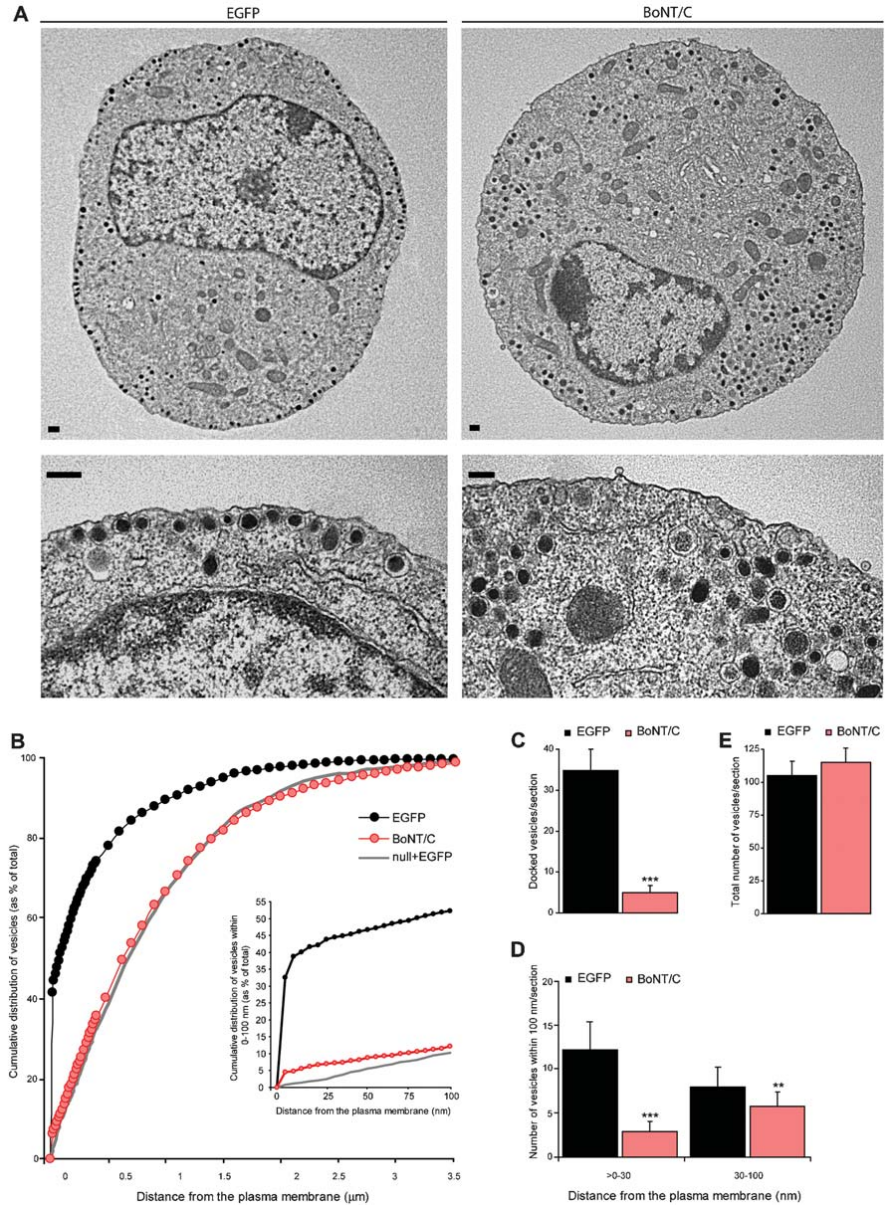
Syntaxin deletion decreases the number of morphologically docked secretory vesicles. (A) Electron micrographs of control and BoNT/C expressing chromaffin cell. For each cell a magnification of a sub-membrane region is shown indicating severely impaired vesicle docking after acute BoNT/C expression compared to the control cell that contains many morphologically docked vesicles at the plasma membrane. Scale bars represent 200 nm. (B) Normalized cumulative distribution of secretory vesicles as a function of distance from the plasma membrane in control cells expressing EFGP or BoNT/C. Inset shows cumulative vesicle distribution in the sub-membrane region within 0–100 nm. Grey line represents the vesicle distribution in the absence of Munc18-1 as shown before [24]. (C–E) Number of docked vesicles (C), vesicles>0–30 and within 30–100 nm (D), and the total number of vesicles (E). Data are mean±SEM from the following number of cells (n) and animals (N): control+EGFP, n = 20, N = 4; control+BoNT/C, n = 20, N = 4 (**p<0.05 and ***p<0.001, ANOVA and student's t-test).

**Figure 3:**
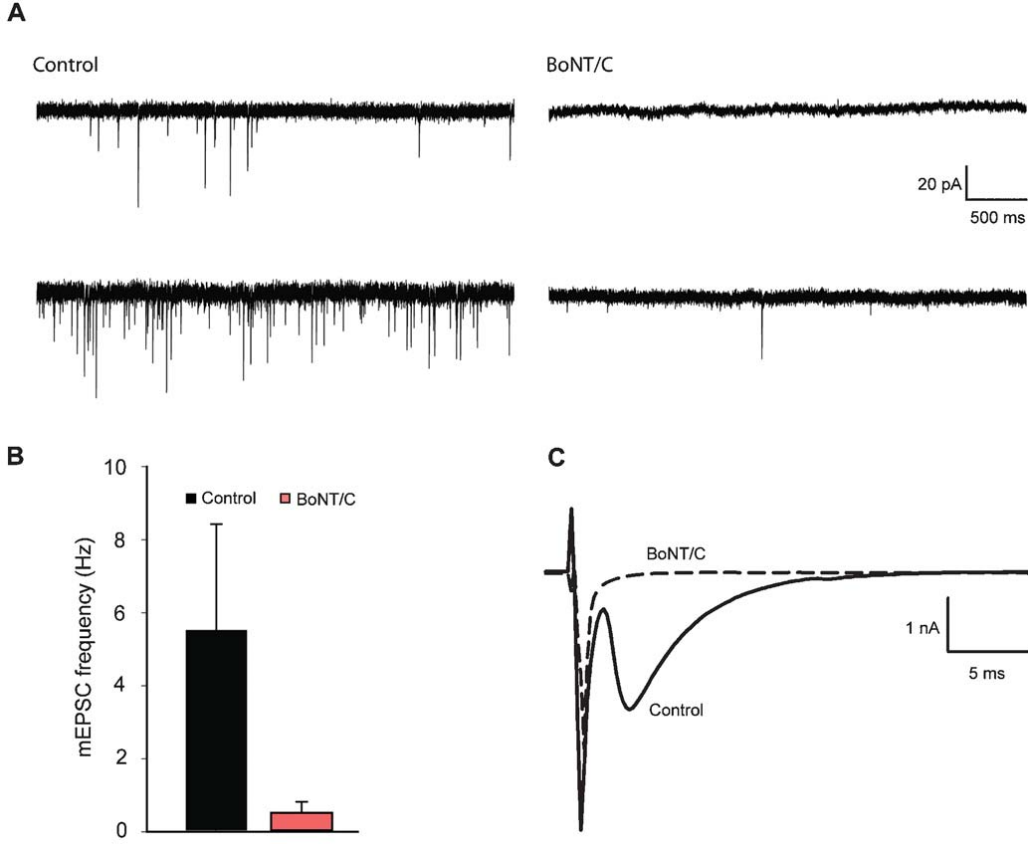
Spontaneous and evoked vesicle fusion is impaired in synapses lacking syntaxin. (A) Representative traces of mEPSC's in whole-cell voltage clamp recordings from control synapses showed frequent spontaneous miniature events, while syntaxin deleted synapses show a strong reduction of spontaneous release. (B) Frequency of spontaneous synaptic events. Numbers indicate mean±SEM for control (n = 5) and BoNT/C infected (n = 4) neurons from N = 2 different animals (***p<0.05, ANOVA and student's t-test). (C) Action potential triggered release is completely blocked by BoNT/C.

**Figure 4:**
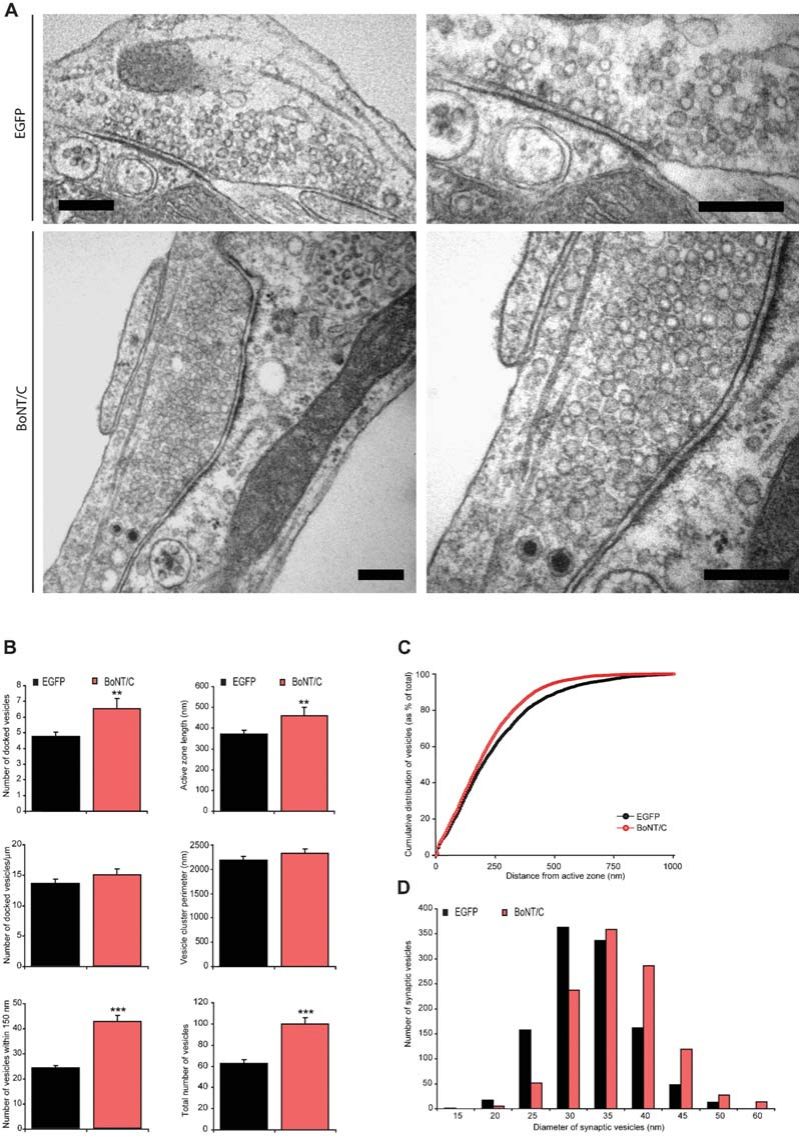
Docking of synaptic vesicles is not impaired after syntaxin proteolysis. (A) Electron micrographs of typical autaptic hippocampus synapse from wild-type autaptic neurons without or with BoNT/C expression. For both conditions a magnification of the same synapse is shown on the right. Scale bars represent 200 nm. Hippocampus autaptic neurons were analyzed after 16 days in culture and 6 hours after infection with SFV. (B) The number of vesicles docked at the active zone is increased after syntaxin cleavage (control 4.8±0.3, n = 62, N = 4 and control+BoNT/C 6.5±0.6, n = 46, N = 4; p<0.05). In the absence of syntaxin the size of the active zone also increased (p<0.05), therefore the number of docked vesicles per active zone length is not changed (p>0.1). The vesicle cluster perimeter do not significantly change (p>0.1), while the number of vesicles within 150 nm from the active zone (control 24.4±0.9; control+BoNT/C 42.8±2.5; p<0.001) as well as the total number of vesicles per synapse is higher in SFV BoNT/C expressing synapses compared to control (control 62.6±3.8; control+BoNT/C 99.9±6.1; p<0.001). Data shown are mean values±SEM (**p<0.05 and ***p<0.001, ANOVA and student's t-test, comparison to control). (C) Normalized cumulative distribution of synaptic vesicles as a function of distance from the plasma membrane in control cells expressing EGFP or BoNT/C. (D) Frequency distribution of the diameter of synaptic vesicles showing a shift towards larger vesicles after syntaxin deletion (ANOVA p<0.001 for n = 1099 vesicles in control n = 62 and control+BoNT/C n = 46 synapses from N = 4 animals).

**Figure 5:**
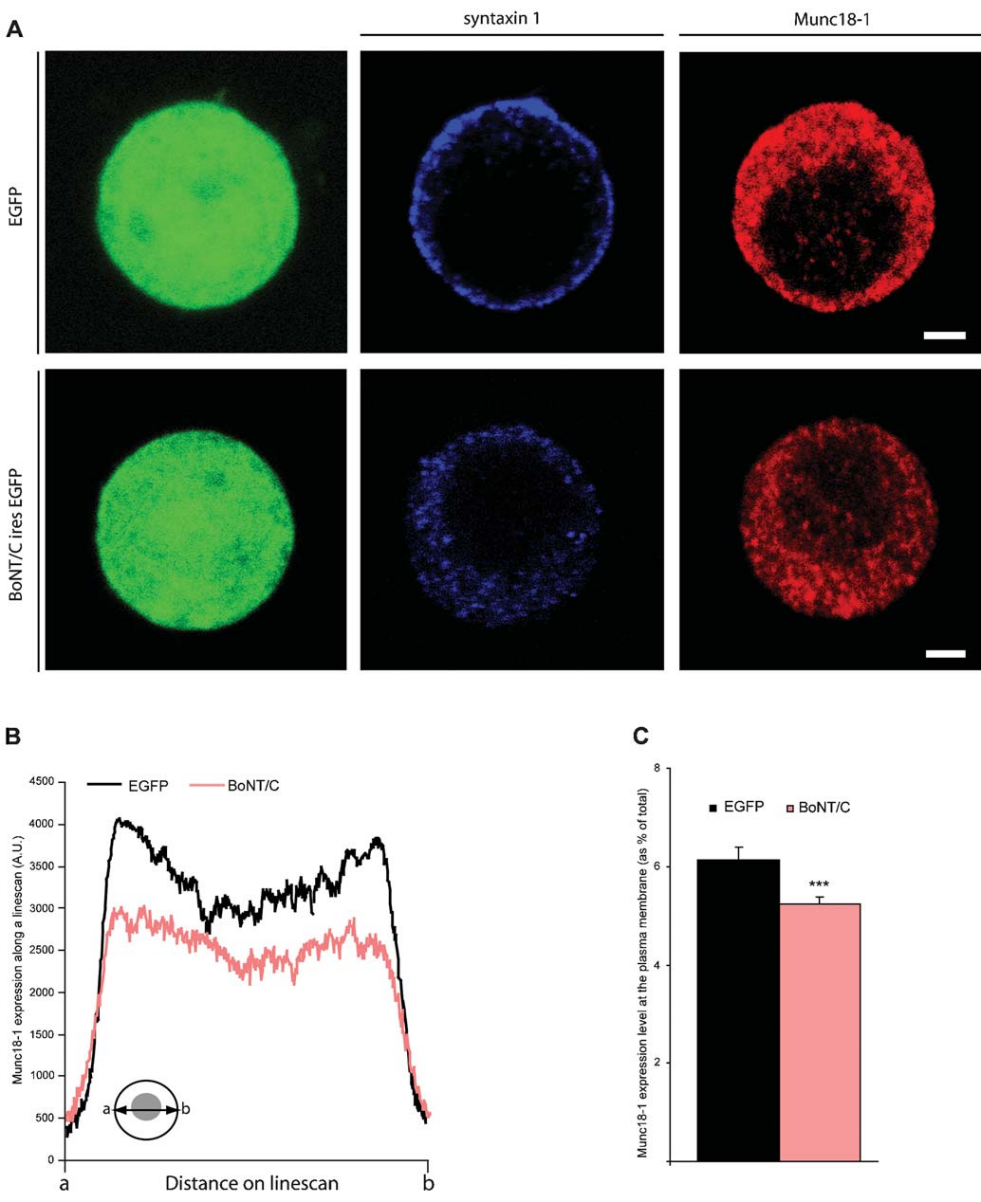
Distribution of Munc18-1 is altered in syntaxin deleted chromaffin cells. (A) Immunolocalization of syntaxin (blue) and Munc18-1 (red) in SFV-*egfp* or *BoNT/C*-ires-*egfp* infected chromaffin cells. Scale bars represent 2 µm. (B) Average pixel intensity of Munc18-1 expression obtained from line scans through a confocal section of a BoNT/C and EGFP expressing cell. Inset shows how line scans were made from **a** to **b** (C) Quantification of the Munc18-1 expression at the plasma membrane. Numbers indicate mean±SEM. from n = 22 cells and N = 3 animals (***p<0.01, ANOVA and student's t-test, comparison to control).

We examined the effect of syntaxin deletion on secretory vesicle docking using morphometric analyses of electron micrographs. In control chromaffin cells, large numbers of secretory vesicles were morphologically docked to the plasma membrane in ultrathin sections (Figure 2A and B) [24]. After syntaxin deletion, a severe reduction of these morphologically docked secretory vesicles was observed (Figure 2A–C). This docking defect was not due to a decreased biogenesis of secretory vesicles, because the total number of vesicles (Figure 2E) was unaffected. The average vesicle diameter was also normal (control: 85.4±11.0 nm, control+BoNT/C: 87.3±9.6 nm; ANOVA p>0.01 for respectively n = 561 and n = 548 vesicles in N = 4 animals and n = 20 cells). A comparison between vesicle distribution in BoNT/C expressing chromaffin cells with *munc18-1* null chromaffin cells [24] (grey line in Figure 2B) indicates that syntaxin deletion produces an exact phenocopy of munc18-1 deletion (Figure 2B). A small but significant difference between syntaxin and munc18-1 deletion was observed in the amount of secretory vesicles within 0–100 nm from the plasma membrane (inset of Figure 2B; ANOVA p<0.05). The subset of secretory vesicles in this 100 nm region of the plasma membrane is thought to represent the morphological correlates of unprimed vesicles [14]. Syntaxin deletion resulted also in a significant decrease of secretory vesicles<30 nm distance from the plasma membrane (Figure 2D; ANOVA, p<0.001), that might represent a separate vesicle pool [14]. These data indicate that syntaxin is involved in secretory vesicle docking in chromaffin cells.

### Docking of synaptic vesicles is not affected after BoNT/C expression

Earlier studies demonstrated that in synapses syntaxin cleavage does not affect synaptic vesicle docking [4], [29]. To elucidate the apparent contrast with chromaffin cells, we expressed BoNT/C in cultured autaptic hippocampus neurons at DIV16. BoNT/C infected and control neurons maintained a dense network of synapses on the timescale of this experiment No other morphological changes were observed in the BoNT/C infected cultures (neurite length, branching, not shown). To confirm that synaptic transmission was silenced in BoNT/C expressing neurons, we examined synaptic function by measuring spontaneous miniature (‘minis’) and evoked excitatory currents. The spontaneous release frequency was strongly reduced but not completely blocked in autaptic hippocampus neurons infected with BoNT/C (Figure 3A and B; control n = 5, control+BoNT/C n = 4) as observed before in rat neurons [25], [30]. Consistent with previous studies [4], [29], action potential triggered release is completely abolished in the BoNT/C infected cells (Figure 3C; excitatory postsynaptic current (EPSC) amplitude in control cells was 2294±514 pA, n = 5, in BoNT/C transfected cells EPSC amplitude could not be detected, n = 4).

Next, we examined the synaptic ultrastructure in BoNT/C expressing autaptic neurons. The overall synapse morphology was not altered (Figure 4A). At low magnification the general appearance of synapses infected with SFV BoNT/C was similar to controls. The ultra structure of asymmetrical synapses was also unchanged, showing docked vesicles in immediate contact with the active zone membrane, many synaptic vesicles in the periphery of the active zone, and a post-synaptic density (Figure 4A). Instead of a docking defect, we observed an increase of docked vesicles at the active zone (Figure 4B), as previously observed in the *Drosophila* neuromuscular junction [5] and giant synapses of squid [29]. The length of the active zone was also increased, and the number of docked vesicles per active zone length was unaltered (Figure 4B). Thus, in contrast to chromaffin cells, synapses do not show a synaptic vesicle docking defect after syntaxin proteolysis. We also quantified the number of vesicles within 150 nm (approximately 3 times the synaptic vesicle size) from the active zone, and the total number of vesicles present in the synapse, and observed for both vesicle populations a significant increase (Figure 4B), consistent with previous observations [3], [29]. No significant difference in the cumulative vesicle distribution was observed (Figure 4C).The synaptic vesicle cluster perimeter did not significantly differ between control and BoNT/C expressing synapses (Figure 4B), while the mean synaptic vesicle diameter was also unchanged (control: 30.7±0.7 nm, control+BoNT/C: 33.9±0.9 nm; respectively n = 1222 and n = 1099 vesicles quantified in n = 62 and n = 46 synapses from N = 4 animals). Within the synaptic vesicle pool we observed an increased number of larger (>45 nm) synaptic vesicles (control: 5.5% and control+BoNT/C: 14.7%; ANOVA p<0.001 for n = 1099 vesicles in n = 62 and n = 46 synapses from N = 4 animals) in syntaxin-deleted synapses (Figure 4D). The larger synaptic vesicles were observed throughout the synapse also in direct contact with the active zone membrane (Figure 4A). A similar phenotype was detected in *Drosophila* strains lacking syntaxin [5].

### Syntaxin deletion results in reduced expression of Munc18-1 at the plasma membrane

Expression of BoNT/C shows the same docking defect as we previously found with Munc18-1 deletion [20], [24]. An impaired targeting or local accumulation of Munc18-1 at docking sites may therefore explain the docking defect in syntaxin-deleted chromaffin cells. Here, we performed a global analysis of the cellular distribution of Munc18-1 within the entire cell. We assumed that the cellular distribution of Munc18-1 along the entire cell diameter is similar to specialized cell areas like cell-cell contacts or areas of substrate contact (‘footprints’), and therefore Munc18-1 localization in these specialized cell areas was not analyzed. Examination of the subcellular localization of Munc18-1 using immunofluorescence staining revealed a punctate labeling in the cytoplasm and discrete puncta at the plasma membrane. This distribution was similar in the presence or absence of syntaxin (Figure 5A). Quantification of Munc18-1 staining revealed indeed a significant, but small reduction of the total average intensity (14.8%, ANOVA p<0.05 for n = 22 cells from N = 2 animals) and plasma membrane staining in BoNT/C expressing cells (14.9%, ANOVA p<0.01; Figure 5B and C). These small reductions in Munc18-1 levels appear to be insufficient to explain the drastic docking defect.

### No alteration of the actin cortex were observed to explain docking defects

**Figure 6:**
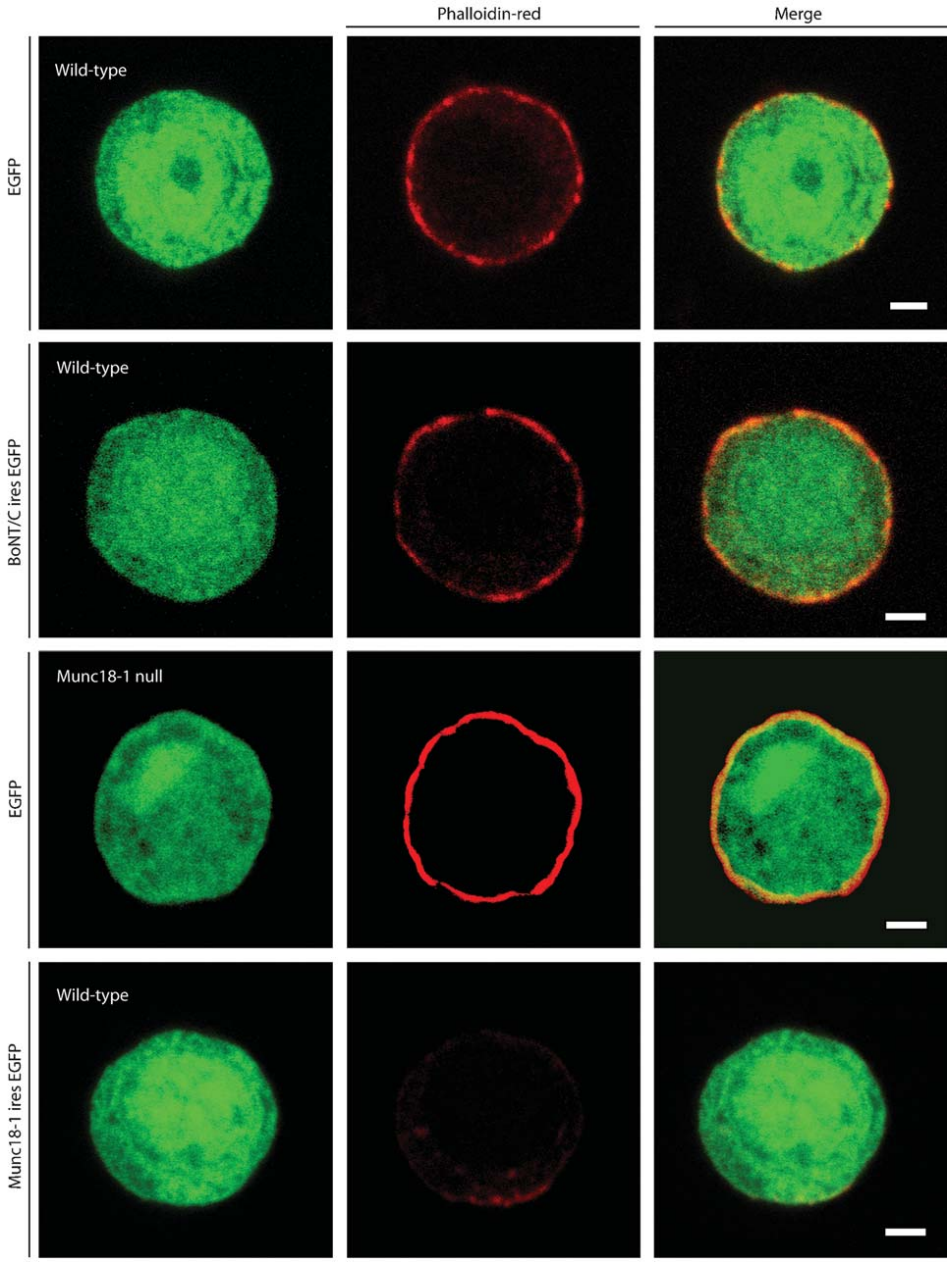
Deletion of syntaxin does not influence the intactness of the sub-membranous actin cytomatrix. Phalloidin-red staining of wild-type chromaffin cells infected with SFV-*egfp, BoNT/C*-ires-*egfp*, or *munc18-1*-ires-*egfp*. As a control Phalloidin-red staining of a *munc18-1* deficient chromaffin cell expressing EGFP is also shown. Merged pictures are shown in the right column. Scale bars represent 2 µm. The data in the lower half of the figures are similar to data published before [24] and are shown here for comparison.

Changes in the sub-membrane actin cortex of secretory cells influence the number of docked secretory vesicles [31]. Previously we demonstrated that Munc18-1 thinners and fenestrate the F-actin cortex and hereby regulates the vesicle ‘hit-rate’ at the target [24]. Since syntaxin is a major binding partner of Munc18-1, we evaluated whether syntaxin proteolysis has similar effects. We visualized the actin cytoskeleton using rhodamin-phaloidin. Munc18-1 under- or overexpression increased or reduced respectively the amount of actin in the F-actin cortex as published before [24], but the intensity/intactness of the sub-membranous actin was similar in the presence or absence of syntaxin (Figure 6). We conclude that syntaxin, unlike its binding partner Munc18-1, does not influence docking by changing the organization of the actin cortex.

## Discussion

In the present study we deleted syntaxin via expression of the light chain of BoNT/C in chromaffin cells and neurons. After syntaxin proteolysis, docking of secretory vesicles in chromaffin cells was strongly reduced, but synaptic vesicle docking was unaltered. Deletion of syntaxin resulted in an exact phenocopy of *munc18-1* null phenotype [20], [24]. These findings are in line with our previous postulate that the syntaxin-Munc18-1 dimer forms a docking platform for secretory vesicles. The observed effects of syntaxin proteolysis on docking may in part be indirect, by promoting targeting and local accumulation of docking factors, such as Munc18-1, at putative docking sites. Munc18-1 cellular levels and its accumulation at the target membrane were reduced after syntaxin cleavage (Figure 5) and expression of Munc18-1 mutants with reduced syntaxin-affinity reduced its plasma membrane association [22]. Furthermore, deletion of a single copy of the syntaxin 4 gene produced a 40% decrease in cellular Munc18-c levels in several non-neuronal tissues [32]. However, the reductions in Munc18-1 level and localization observed in the present study were small and are unlikely to explain the complete docking defect. We also observed a small difference between Munc18-1 and syntaxin mutants in a subset of secretory vesicles within 100 nm. This difference could either reflect separate roles of each protein like shown for UNC-10 (RIM) and UNC-13 [14], different tethering modes [24], or represents complexes of syntaxin protected from BoNT/C proteolysis [33]. Therefore, we conclude that syntaxin directly promotes docking in secretory cells, probably in conjunction with Munc18-1. Alternatively, syntaxin may also promote docking by establishing a synaptotagmin binding site in a heterodimer with SNAP-25 [10], [15], [27], [34]. Syntaxin may also regulate docking by reducing the actin network barrier, similar to Munc18-1 [24] or by promoting actin-based vesicle transport via myosin-V [35]. However, we did not observe changes in the actin network upon syntaxin deletion (Figure 6). Hence, the function of Munc18-1 in actin network regulation appears to be syntaxin-independent.

BoNT/C also cleaves SNAP-25, albeit with a much lower efficiency [36]. Recently, it was shown that shortening of the SNAP-25 C-terminal tail by BoNT/A (9-amino acids) reduces the thermo stability of SNARE complexes [37]and results in a concomitant reduction of the ready releasable pool [38]. Docking was not analyzed in these studies, but docking was not affected in SNAP-25 deficient chromaffin cells [8]. Therefore, it is unlikely that SNAP-25 proteolysis contributes to the docking effect upon BoNT/C.

We found no evidence for a docking role of syntaxin in synapses, in line with earlier studies [4], [5], [29]. A possible explanation for the difference between chromaffin cells and nerve terminals is that neurons may have evolved a separate, SNARE-independent docking mechanism as a specialization for rapid and regulated membrane fusion. However, given the overwhelming evidence for a high degree of conservation in vesicle trafficking principles from yeast to human, especially for SNARE dependent mechanisms [1], [2], it seems more likely that in neurons additional layers of regulation have evolved to control docking and to accommodate the specific features of synaptic transmission. Such additional factors may have rendered syntaxin's docking role redundant. Neuron-specific scaffolding proteins, such as bassoon, RIM, piccolo and Bruchpilot may be responsible for such additional regulation. These are large proteins that accumulate at the active zone, determine structural and functional properties of the terminal and for instance control clustering of Ca^2+^-channels [39]–[42]. Indeed, UNC10 (RIM) deletion in nematodes or its upstream effector, Rab3, result in a partial loss of docked vesicles [13], [14]. This partial loss of docking is consistent with the idea of multiple docking pathways in synapses. Docking sites for secretory vesicles, on the other hand, appear to be less complex. Most of the active zone-scaffolding proteins are not expressed and there is more free space surrounding docked vesicles. The undocking effect of a putative docking factor like syntaxin is expected to be much larger and evident using morphological assays.

An alternative explanation for the fact that synapses show no docking defect after BoNT/C cleavage may be that other non-cognate SNAREs that normally do not function in docking substitute for the deleted syntaxins, like proposed for exocytosis [6]. However, all major plasma membrane syntaxins, syntaxin 1–3, are cleaved by BoNT/C and the only known resistant paralog, syntaxin 4 [43], is expressed only at low levels and in specific synapses [44].

Synaptic vesicles had, on average, a larger diameter after BoNT/C expression. Larger synaptic vesicles were also observed after genetic deletion of syntaxin in flies [5] and in synaptobrevin deficient murine synapses [45]. This effect is reminiscent to earlier observations in mutants of clathrin adaptor proteins in flies and nematodes [46], [47]. As this effect appears to be consistent among SNARE-deficient and endocytosis-compromised systems, increased vesicle diameter may be a general consequence of vesicle cycle arrest.

## Material and Methods

### Cell culture and Infection

Embryonic (E18) mouse chromaffin cells were prepared as described [8] and experiments performed on the 2^nd^–4^th^ day after isolation. Microisland hippocampus cultures were prepared from wild-type mouse embryos at E18 according to [48] and experiments performed at DIV16. Acute expression of heterologous genes was induced using Semliki Forest Virus (SFV). Genes of interest were expressed from a bi-cistronic message containing *egfp* [20], [24]. Experiments were performed after 6 hr of infection. BoNT/C light chain (kind gift from T Galli, INSERM, Paris, France) and Munc18-1 have been described before [24]. Constructs were verified by sequencing.

### Electron microscopy

Chromaffin cells from wild type or *munc18-1* deficient (E18) mice were plated on rat tail type 1 collagen-coated (32 µg/ml; Beckton Dickinson labware, USA) Bellco gridded glass coverslips (Bellco Glass Inc., USA) and infected (DIV2) with *BoNT/C*-ires-*egfp, munc18-1*-ires-*egfp*, or SFV-*egfp* as a control. Cells were observed under a fluorescence microscope 6h after infection and the location of infected/control cells were mapped. At the time secretion was blocked in BoNT/C expressing cells, cells were fixed for 45 min at room temperature with 2.5% glutaraldehyde in 0.1 M cacodylate buffer (pH 7.4). After fixation cells were washed three times for 5 min with 0.1 M cacodylate buffer (pH 7.4), post-fixed for 2 hr at room temperature with 1% OsO_4_ in bidest, washed and stained with 1% uranyl acetate for 40 min in the dark. Following dehydration through a series of increasing ethanol concentrations, cells were embedded in Epon and polymerized for 24 h at 60°C. The coverslip was removed by alternately dipping in liquid nitrogen and hot water. Cells of interest were selected by observing the flat Epon embedded cell monolayer (containing the gridded Bellco print) under the light microscope, and mounted on pre-polymerized Epon blocks for thin sectioning. Ultra thin sections (∼90 nm) were cut parallel to the cell monolayer and collected on single-slot, formvar-coated copper grids, and stained with uranyl acetate and lead citrate. For each condition the relative frequency of docked vesicles, and vesicles within 30 or 100 nm from the plasma membrane were calculated in three different grids per animal in a JEOL 1010 electron microscope. Docked vesicles were without any measurable distance between granule and plasma membrane. Distances from the granule membrane to the plasma membrane were measured on digital images taken at 20.000× magnification using analySIS software (Soft Imaging System, Gmbh, Germany). Secretory vesicles were recognized by their round, dense core and had a diameter of approximately 90 nm. The observer was blinded for the genotype.

Hippocampus islands cultures of wild-type mice (E18) were grown on Bellco gridded coverslips that contain micro islands of glia cells. Wild-type hippocampus neurons were infected (DIV16) with *BoNT/C*-ires-*egfp* or SFV-*egfp* as control and observed under a fluorescence microscope 6h after infection to map the location of infected cells. Only glia islands containing a single neuron were used for analysis. Fixation was performed at the time when evoked-release was blocked in BoNT/C expressing neurons. Fixation, embedding and sectioning were the same as for chromaffin cells (see above). Autaptic synapses were selected at low magnification using a JEOL 1010 electron microscope. The distribution of synaptic vesicles, total vesicle number, size of the vesicle cluster, post synaptic density and active zone length were measured on digital images taken at 100.000× magnification using analySIS software (Soft Imaging System, Gmbh, Germany). The observer was blinded for the genotype. No difference was observed in any of the parameters measured between wild-type synapses expressing SFV-*egfp* and non-infected wild-type synapses, these synapses were therefore pooled.

### Immunofluorescence microscopy

Mouse chromaffin cells were infected (DIV2) with SFV-*egfp, BoNT/C*-ires-*egfp* or *munc18-1*-ires-*egfp* and fixed after 6 hr in 4% paraformaldehyde, permeabilized in 0.1% Triton X-100 and blocked with 2% goat serum. Cells were incubated with primary antibody (anti-Munc18-1, polyclonal #2701 produced in our laboratory; anti-syntaxin1, monoclonal #HPC1, Sigma), washed and stained with the secondary antibody (goat anti-rabbit Alexa 594 or goat anti-mouse Alexa 647 or 594). Filamentous actin was stained by incubation with 0.25 U/ml rhodamin-phalloidin (Molecular Probes) in PBS for 40 min. As a control filamentous actin was stained in *munc18-1* null chromaffin cells infected with *SFV*-*egfp*. In each experiment, coverslips were viewed with a 63× objective Zeiss LSM510 fluorescence microscope and confocal images were acquired using identical photomultiplier settings and corrected for background fluorescence using unlabelled specimens. The Munc18-1 expression level on the plasma membrane was determined along line scans of the entire cell diameter using the Metamorph software (Universal Imaging Corporation, West Chester, USA) and normalized to the total intensity.

### Electrophysiology

Carbon-fiber amperometry on mouse chromaffin cells were performed 6h after infection at 30–32°C. Single-stranded insulated carbon fibers (diameter 6 µm, model CC-18, van den Hul, Oene, The Netherlands) were mounted in glass micro capillaries (GC150-10, Harvard Apparatus Ltd, Kent, UK). GigaOhm resistance (2–5 GW) to ground was achieved by insulating the microelectrode and carbon fiber with Sylgard. The tip of the carbon fiber was cut just before the experiment to ensure cleanliness and sensitivity of the exposed tip surface. Microelectrodes were filled with 1 M KCl and placed in close apposition to the cell surface. Amperometric currents were recorded with an EPC8 amplifier (HEKA Electronics, Lambrecht, Germany; electrode voltage set to +650 mV), sampled at 10 kHz and filtered at 3 kHz. Release was evoked using high 30 nM K^+^ solution.

Neurons were infected 6 hours before electrophysiological recordings with Semliki Forest virus. Whole cell voltage-clamp recordings were performed on cultured hippocampus autaptic neurons between DIV 15 and 16. The patch pipette contained the following solution (in mM): 125 K^+^-gluconic acid, 10 NaCl, 4.6 MgCl_2_, 4 K_2_-ATP, 15 creatine phosphate, 1 EGTA and 20 U/ml phospocreatine kinase (pH 7.30). External medium contained (in mM): 140 NaCl, 2.4 KCl, 4 CaCl_2_, 4 MgCl_2_, 10 HEPES, 10 glucose (pH 7.30). Axopatch 200A (Axon Instruments, Union City, USA) was used for whole-cell recordings and signals were acquired using Digidata 1322A and Clampex 8.1 (Axon Instruments). Clampfit 8.0 (Axon Instruments) and Mini Analysis (Synaptosoft) was used for offline analysis. All experiments were conducted at 31°C.

### Statistical analysis

Data are shown as mean values±SEM Statistical significance was determined by comparison between experiments (means of all chromaffin cells or synapses within an experiment) using paired student's t-test or analysis of variance.
